# The mRNA–miRNA–lncRNA Regulatory Network and Factors Associated with Prognosis Prediction of Hepatocellular Carcinoma

**DOI:** 10.1016/j.gpb.2021.03.001

**Published:** 2021-03-17

**Authors:** Bo Hu, Xiaolu Ma, Peiyao Fu, Qiman Sun, Weiguo Tang, Haixiang Sun, Zhangfu Yang, Mincheng Yu, Jian Zhou, Jia Fan, Yang Xu

**Affiliations:** 1 Department of Liver Surgery and Transplantation, Liver Cancer Institute, Zhongshan Hospital, and Key Laboratory of Carcinogenesis and Cancer Invasion (MOE), Fudan University, Shanghai 200032, China; 2 State Key Laboratory of Genetic Engineering, Fudan University, Shanghai 200032, China; 3 Institute of Biomedical Sciences, Fudan University, Shanghai 200032, China; 4 Laboratory Medicine Department, Zhongshan Hospital, and Key Laboratory of Carcinogenesis and Cancer Invasion (MOE), Fudan University, Shanghai 200032, China; 5 Institute of Fudan-Minhang Academic Health System, Minhang Hospital, Fudan University, Shanghai 201199, China

**Keywords:** TCGA database, mRNA–miRNA–lncRNA regulatory network, Hepatocellular carcinoma, Prognostic factor, Systems biology

## Abstract

The aim of this study was to identify novel prognostic mRNA and microRNA (miRNA) biomarkers for **hepatocellular carcinoma** (HCC) using methods in **systems biology**. Differentially expressed mRNAs, miRNAs, and long non-coding RNAs (lncRNAs) were compared between HCC tumor tissues and normal liver tissues in The Cancer Genome Atlas (TCGA) database. Subsequently, a prognosis-associated mRNA co-expression network, an mRNA–miRNA regulatory network, and an **mRNA–miRNA–lncRNA regulatory network** were constructed to identify prognostic biomarkers for HCC through Cox survival analysis. Seven prognosis-associated mRNA co-expression modules were obtained by analyzing these differentially expressed mRNAs. An expression module including 120 mRNAs was significantly correlated with HCC patient survival. Combined with patient survival data, several mRNAs and miRNAs, including *CHST4*, *SLC22A8*, *STC2*, hsa-miR-326, and hsa-miR-21 were identified from the network to predict HCC patient prognosis. Clinical significance was investigated using tissue microarray analysis of samples from 258 patients with HCC. Functional annotation of hsa-miR-326 and hsa-miR-21-5p indicated specific associations with several cancer-related pathways. The present study provides a bioinformatics method for biomarker screening, leading to the identification of an integrated mRNA–miRNA–lncRNA regulatory network and their co-expression patterns in relation to predicting HCC patient survival.

## Introduction

Hepatocellular carcinoma (HCC) is the third leading cause of cancer-related deaths in the world, accounting for approximately 662,000 deaths per year [1]. Only 10%–20% of HCCs are surgically resectable [2]. Although etiological factors, including alcohol, hepatitis B/C virus, and aflatoxin B1, have been identified, the underlying molecular pathogenesis of HCC remains poorly understood [3].

Previous studies have demonstrated that growth factors, such as transforming growth factor-α (TGF-α) and TGF-β [4], [5], and tumor suppressor genes, such as *RB* and *TP53*, are implicated in the development of HCC [6], [7]. Recent genomic profiling studies have provided new insights into molecular hepatocarcinogenesis [8] and indicated altered Wnt/β-catenin and JAK1/STAT signaling [8], [9]. Moreover, profiling of microRNAs (miRNAs) and long non-coding RNAs (lncRNAs) has identified specific miRNAs and lncRNAs involved in HCC carcinogenesis [10], [11]. For example, miR-24 promoted aflatoxin B1-related HCC and may, therefore, be used to predict patient prognosis [12]. Dysregulated miR-150-5p, miR-195-5p, miR-21, miR-221-3p, and miR-224-5p, as well as lncRNAs *UCA1*, *MALAT-1*, and *HOTAIR*, also play important roles in HCC [13], [14]. Although progress has been made, comprehensive understanding of HCC carcinogenesis and prognosis is lacking.

A recent study has reported that mRNAs, miRNAs, and lncRNAs interdependently regulate HCC pathogenesis [15]. However, to our knowledge, there are relatively few studies examining the role of the mRNA–miRNA–lncRNA regulatory network in HCC prognosis. The aim of the present study was to explore a novel mRNA–miRNA–lncRNA regulatory network to identify prognostic mRNA and miRNA biomarkers for HCC. Based on RNA-seq and miRNA-seq data, we evaluated differentially expressed mRNAs (DEmRNAs) and differentially expressed miRNAs (DEmiRNAs) in HCC tumor tissues compared with normal liver tissues. By combining our results with patient prognosis information obtained from The Cancer Genome Atlas (TCGA) database, we identified key nodes in the DEmiRNA–mRNA regulatory network that may predict HCC patient prognosis. The prognosis-associated mRNA co-expression modules and the DEmiRNA–mRNA regulatory network were constructed to further identify prognostic biomarkers for HCC. Two miRNAs, hsa-miR-326 and hsa-miR-21, and three mRNAs, *CHST4*, *SLC22A8*, and *STC2*, were found to be strong predictors of HCC prognosis. We confirmed the results from our network analysis in a clinical cohort of 50 cases of HCC. In summary, this study reports a novel method of cancer biomarker identification by combining miRNA, lncRNA, and mRNA data, providing critical insights about HCC development.

## Results

### Differential expression and functional enrichment of mRNAs

**Figure 1:**
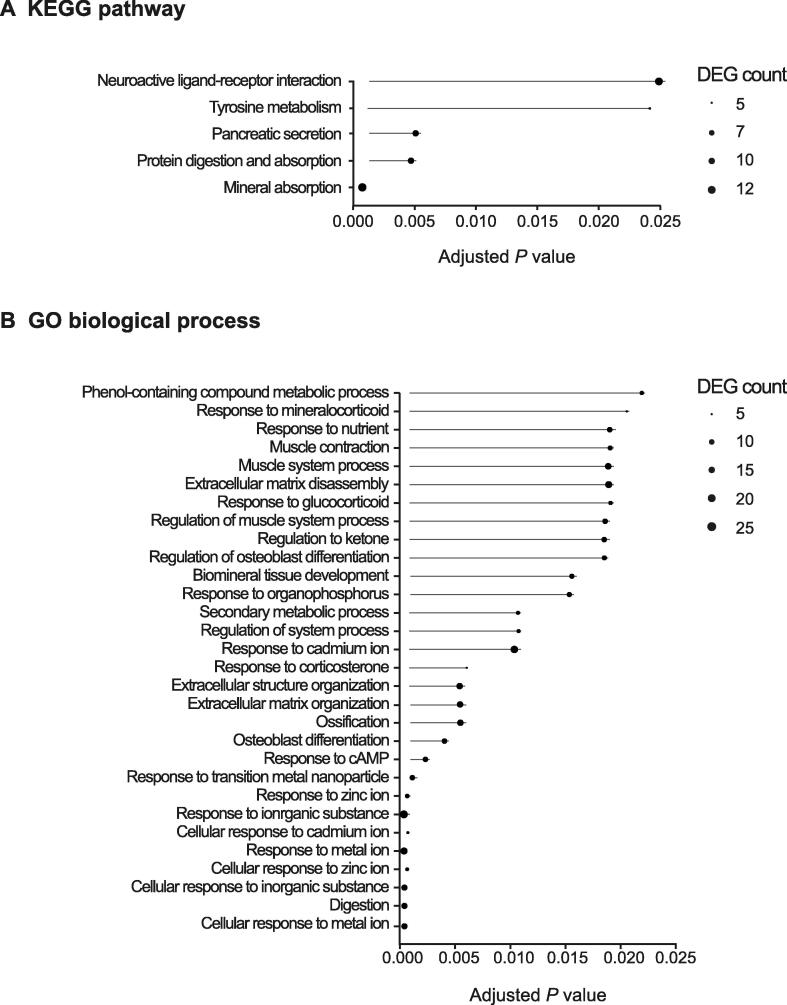
**KEGG pathway and GO biological process enrichment analyses of DEmRNAs in HCC tumors** **A.** KEGG pathways enriched by DEmRNAs in HCC tumors. **B.** Top 30 GO biological processes enriched by DEmRNAs in HCC tumors. KEGG, Kyoto Encyclopedia of Genes and Genomes; GO, Gene Ontology; DEmRNA, differentially expressed mRNA; HCC, hepatocellular carcinoma.

mRNA expression was compared between HCC tumors and normal samples. We identified 399 DEmRNAs, including 272 up-regulated and 127 down-regulated mRNAs. In addition, 1 up-regulated and 5 down-regulated lncRNAs were identified. We also found 233 DEmiRNAs, including 39 up-regulated and 194 down-regulated miRNAs. Kyoto Encyclopedia of Genes and Genomes (KEGG) analysis of DEmRNAs showed significantly enriched pathways involved in mineral absorption, protein digestion and absorption, and tyrosine metabolism (Figure 1A). Gene Ontology (GO) analysis showed that biological processes associated with cellular responses to metal ions and extracellular matrix organization and disassembly were also enriched (Figure 1B). However, conventional enrichment analyses did not yield sufficient information about differences in DEmRNAs between tumor and normal tissues.

### mRNA co-expression network analysis

**Figure 2:**
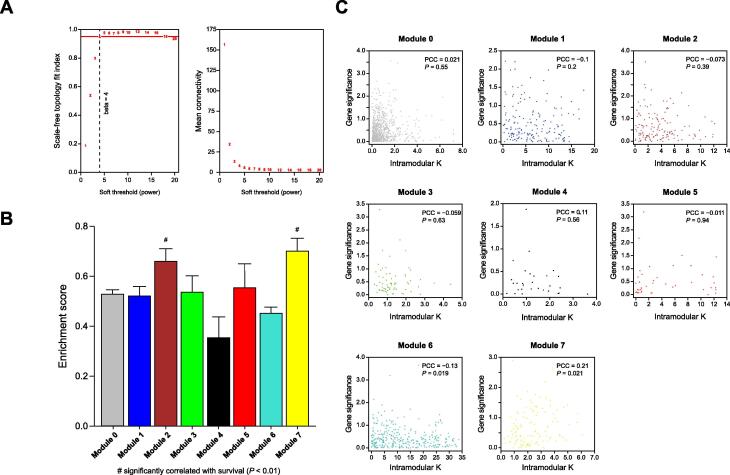
**WGCNA of mRNA co-expression in HCC** **A.** The soft threshold for the construction of the mRNA co-expression network. **B.** Enrichment of survival genes in different modules. Modules 1–7 indicate different co-expressed mRNA patterns identified by WGCNA. Module 0 labeled in gray corresponds to the set of mRNAs which have not been clustered in any module. **C.** mRNA relationships in modules. WGCNA, weighted correlation network analysis; PCC, Pearson correlation coefficient.

In the mRNA co-expression network constructed by weighted correlation network analysis (WGCNA), a scale-free biological network could be constructed with beta = 4 (Figure 2A). Seven modules (Modules 1–7) were identified with the parameters of cutHeight = 0.99 and minSize = 30. These modules are displayed as different colors in a hierarchical clustering diagram. The mRNA with a higher degree (hub mRNA) in the module may have a stronger correlation with disease. Two modules, Module 7 (yellow) and Module 2 (brown), contained hub mRNAs that were significantly correlated with survival (Figure 2B; *P* < 0.01). Indeed, mRNAs in Module 7 and Module 6 (cyan) had significantly more internal interactions (Figure 2C). Module 7 was considered the most significant module, as it included 120 mRNAs. The top 20 mRNAs with the highest intramodular connectivity (*kWithin*) in Module 7 are shown in Table 1. Based on the *P* value of Cox regression, five mRNAs with *P* < 0.05 were considered hub mRNAs, including exportin 5 (*XPO5*), centromere protein H (*CENPH*), peptidylprolyl isomerase-like 1 (*PPIL1*), RNA polymerase II subunit G (*POLR2G*), and bystin-like (*BYSL*).

**Table 1:** **Top 20 mRNAs with the highest intramodular connectivity in Module 7**

**Gene**	***P* value**	***kTotal***	***kWithin***
*CCNB1*	0.371	10.629	6.116
*MRTO4*	0.074	10.633	5.774
*PLK1*	0.141	10.988	5.755
*NUP37*	0.165	9.257	5.162
***XPO5***	**0.038**	**8.123**	**5.031**
*CCNF*	0.536	8.206	4.903
***CENPH***	**0.046**	**7.994**	**4.755**
*RCC1*	0.440	7.683	4.675
*SCAMP3*	0.401	8.932	4.448
*GPN2*	0.353	6.685	4.414
*RRP36*	0.135	7.597	4.267
*BIRC5*	0.056	10.841	4.239
*LEMD2*	0.580	7.867	4.191
*FARSB*	0.107	8.089	4.160
*SLC25A19*	0.077	7.203	3.960
***PPIL1***	**0.002**	**6.295**	**3.925**
*MRGBP*	0.133	9.744	3.921
***POLR2G***	**0.027**	**9.246**	**3.748**
*GPATCH3*	0.438	7.317	3.745
***BYSL***	**0.039**	**6.314**	**3.719**

*Note*: mRNAs are ranked according to the values of *kWithin*, and hub mRNAs are indicated in bold (*P* < 0.05; Cox regression). *kTotal*, intermodular connectivity; *kWithin*, intramodular connectivity.

### Construction of the DEmiRNA–mRNA regulatory network

**Figure 3:**
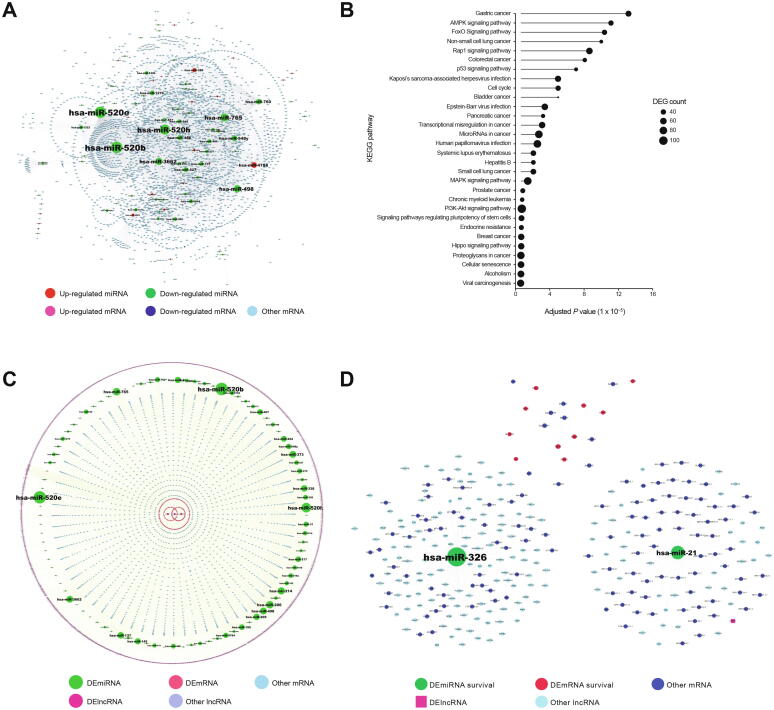
**The mRNA–miRNA–lncRNA regulatory network and survival-associated subnetworks** **A.** The constructed DEmiRNA–mRNA network. **B.** Functional enrichment analysis of DEmiRNAs. **C.** The constructed mRNA–miRNA–lncRNA complex regulatory network. **D.** mRNA–miRNA–lncRNA subnetworks associated with survival. DEmiRNA, differentially expressed miRNA; DElncRNA, differentially expressed lncRNA.

We identified 5558 DEmiRNA–mRNA interaction pairs, including 86 DEmiRNAs and 3377 mRNAs. The DEmiRNA–mRNA regulatory network was constructed using Cytoscape [16] (Figure 3A). The top 20 miRNAs with highest degree or betweenness are shown in Table 2. KEGG analysis revealed that the DEmiRNAs in the DEmiRNA–mRNA regulatory network were significantly enriched for the Hippo, MAPK, and PI3K-Akt signaling pathways (Figure 3B).

**Table 2:** **Top 20 miRNAs with the highest degree or betweenness**

**miRNA**	**Degree**	**miRNA**	**Betweenness**
hsa-miR-520b	423	hsa-miR-224	1
hsa-miR-520e	420	hsa-miR-498	0.118
hsa-miR-520 h	364	hsa-miR-765	0.116
hsa-miR-765	276	hsa-miR-520b	0.114
hsa-miR-498	249	hsa-miR-520 h	0.114
hsa-miR-3662	244	hsa-miR-3662	0.112
hsa-miR-548y	184	hsa-miR-520e	0.105
hsa-miR-4784	182	hsa-miR-548y	0.081
hsa-miR-760	174	hsa-miR-466	0.073
hsa-miR-466	160	hsa-miR-760	0.071
hsa-miR-527	144	hsa-miR-4784	0.064
hsa-miR-1276	141	hsa-miR-527	0.056
hsa-miR-137	122	hsa-miR-137	0.056
hsa-miR-326	118	hsa-miR-1276	0.054
hsa-miR-944	116	hsa-miR-326	0.049
hsa-miR-577	115	hsa-miR-944	0.046
hsa-miR-496	110	hsa-miR-577	0.044
hsa-miR-6844	106	hsa-miR-496	0.043
hsa-miR-1323	104	hsa-miR-599	0.042
hsa-miR-599	102	hsa-miR-6844	0.042

### Construction of the mRNA–miRNA–lncRNA complex regulatory network

**Figure 4:**
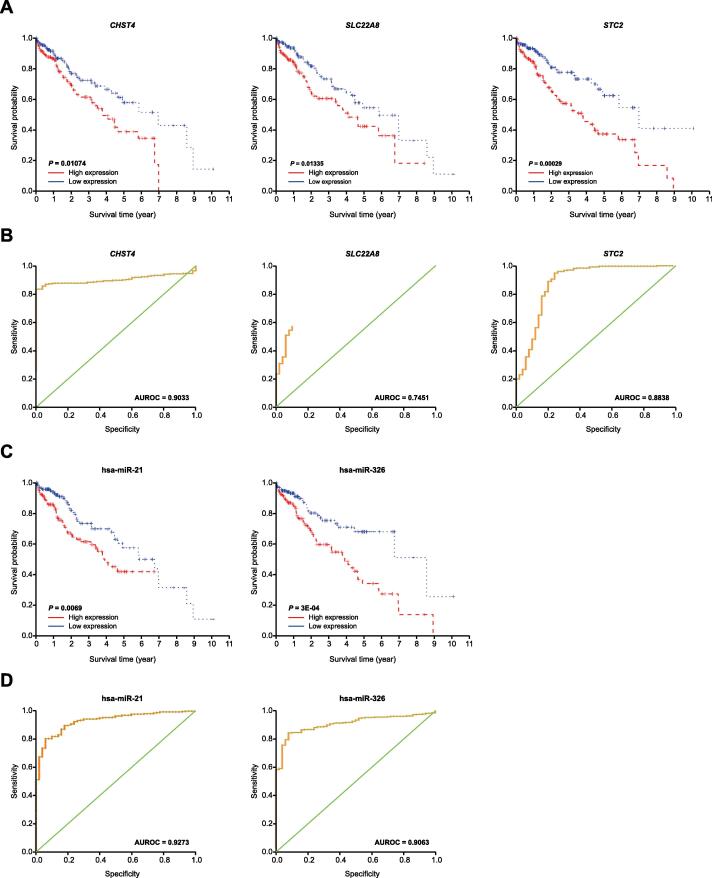
**The potential biomarkers for the prognosis of HCC** **A.** Kaplan-Meier survival curves for HCC patients with different expression levels of *CHST4*, *SLC22A8*, and *STC2*. **B.** ROC curves of *CHST4*, *SLC22A8*, and *STC2* expression for predicting overall survival of HCC patients. **C.** Kaplan-Meier survival curves for HCC patients with different expression levels of hsa-miR-326 and hsa-miR-21. **D.** ROC curves of hsa-miR-326 and hsa-miR-21 expression for predicting overall survival of HCC patients. ROC, receiver operating characteristic; AUROC, area under the ROC curve.

We next identified 331 DEmRNA–lncRNA pairs and 4313 DEmiRNA–lncRNA pairs with significant co-expression (*P* < 0.01). These pairs were combined with the 5558 DEmiRNA–mRNA pairs by merging common nodes, and an mRNA–miRNA–lncRNA complex regulatory network was finally constructed with 4492 nodes and 10,202 interacting pairs (Figure 3C). Furthermore, we identified subnetworks that were significantly correlated with patient survival (Figure 3D). Among these subnetworks, ten mRNAs (*CDH6*, *CHST4*, *CXCL1*, *DNER*, *IL20RB*, *PROK1*, *SBSN*, *SLC22A8*, *STC2*, and *TCN1*) were significantly correlated with HCC patient survival, and their higher expression predicted worse prognosis (Figure 4A, Figure S1A). Based on the support vector machine (SVM), although the model was trained and tested on different data after randomly splitting the dataset into two pieces (10-fold cross-validation), we evaluated the classification effects of the ten mRNAs. *CHST4*, *SLC22A8*, and *STC2* had higher classification effects than other genes (Figure 4B, Figure S1B). In the subnetworks, two miRNAs (hsa-miR-326 and hsa-miR-21) regulated a vast number of lncRNAs and mRNAs (Figure 3D). The higher expression of hsa-miR-326 and hsa-miR-21 predicted worse prognosis for HCC patients (Figure 4C), and they also displayed good classification performance [area under the receiver operating characteristic curve (AUROC) = 0.9063 for hsa-miR-326; AUROC = 0.9273 for hsa-miR-21] (Figure 4D). Consistent with those results, the differentially expressed lncRNA (DElncRNA) *PART1* was regulated by hsa-miR-21 (Figure 3D).

**Figure 5:**
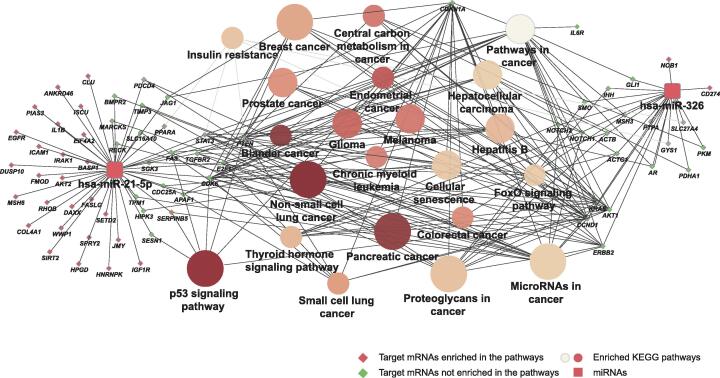
**Target mRNAs and enriched pathways of hsa-miR-326 and hsa-miR-21** miRNA and miRNA target are represented with square and diamond, respectively. Green diamond represents the target enriched in the pathway, and red diamond represents the target that is not enriched in the pathway; circle represents a significantly enriched pathway (the larger the node, the more significant the pathway; the darker the color, the greater the proportion of genes enriched in this pathway).

Next, we performed KEGG pathway enrichment analysis for the target mRNAs of hsa-miR-326 and hsa-miR-21. Our results showed that hsa-miR-21 was associated with the p53 signaling pathway, and hsa-miR-326 was associated with the FoxO signaling pathway (Figure 5).

### Clinical validation of five selected mRNAs/miRNAs

**Figure 6:**
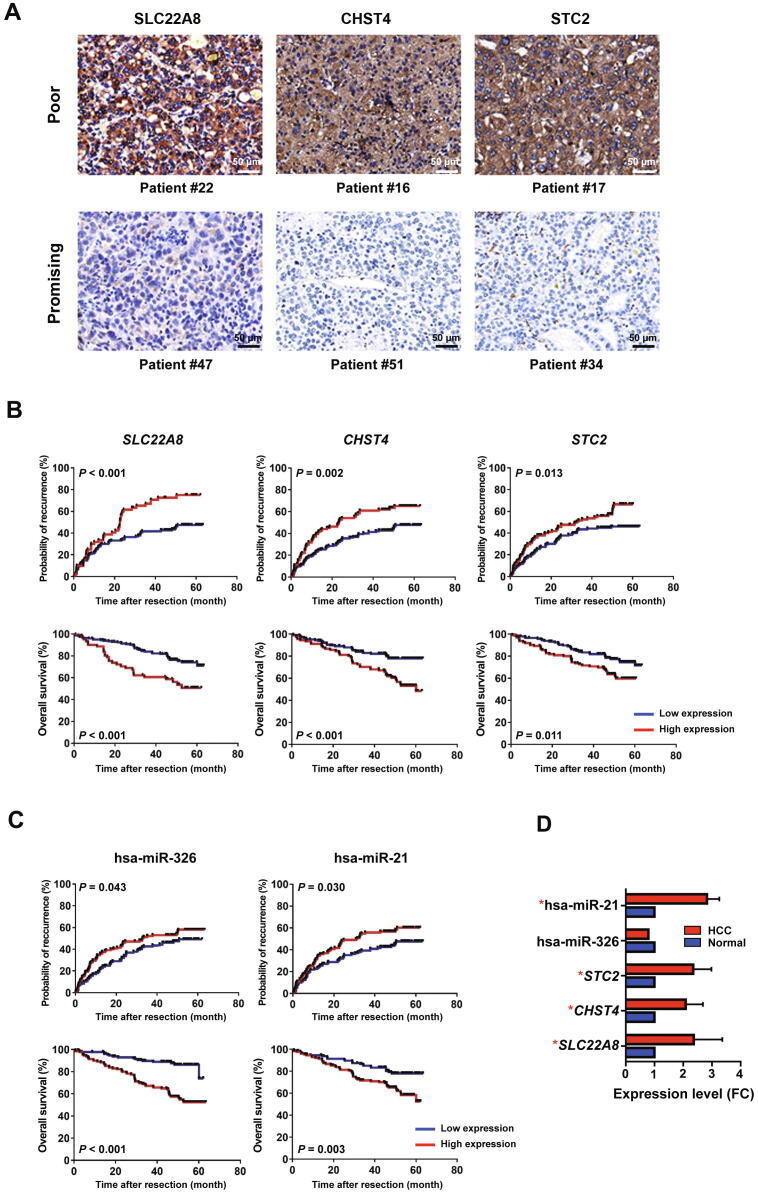
**Clinical significance of *CHST4*, *SLC22A8*, *STC2*, hsa-miR-326, and hsa-miR-21** **A.** Typical images of IHC staining for SLC22A8, CHST4, and STC2 in HCC patients with distinct prognosis. Scale bar, 50 μm. **B.** Clinical validation of the prognostic significance of *SLC22A8*, *CHST4*, and *STC2*. Upper: the significance for predicting recurrence; lower: the significance for predicting overall survival. **C.** Clinical validation of the prognostic significance of has-miR-326 and has-miR-21. Upper: the significance for predicting recurrence; lower: the significance for predicting overall survival. **D.** Expression status of selected mRNAs/miRNAs between HCC and paired adjacent normal liver tissues. The average expression levels of indicated mRNAs/miRNAs in normal tissues were set as 1.0, and the expression levels of mRNAs/miRNAs in HCC tissues were calculated as HCC/normal to determine the FC in expression. IHC, immunohistochemistry; FC, fold change.

Next, we validated the clinical significance of five mRNAs/miRNAs (*SLC22A8*, *CHST4*, *STC2*, has-miR-326, and has-miR-21) selected from our integrated analysis. A tissue microarray representing 258 HCC patients was stained by immunohistochemistry (IHC) using antibodies for CHST4, SLC22A8, and STC2*.* In addition, RT-PCR was performed for hsa-miR-326 and hsa-miR-21. Typical IHC staining images of tissues from patients with different prognoses are shown in Figure 6A. High expression of *CHST4*, *SLC22A8*, and *STC2* predicted shorter time-to-relapse (TTR) and overall survival (OS) in patients with HCC (Figure 6B; *P* < 0.05). Similarly, high expression of hsa-miR-326 and hsa-miR-21 was correlated significantly with poor prognosis (Figure 6C; *P* < 0.05). In addition, we evaluated the expression patterns of the five mRNAs/miRNAs in HCC tissues and paired liver tissues. The expression levels of these mRNAs/miRNAs were significantly higher in HCC tissues than those in paired liver tissues (*P* < 0.05), with the exception of has-miR-326 (Figure 6D). These data suggest that these mRNAs/miRNAs play important roles in HCC pathogenesis and progression.

## Discussion

A novel mRNA–miRNA–lncRNA complex regulatory network associated with HCC prognosis was constructed in the present study. Integrated analysis of this regulatory network identified a module containing *CHST4*, *SLC22A8*, *STC2*, hsa-miR-326, and hsa-miR-21. This module was significantly correlated with HCC patient survival and prognosis. Moreover, hsa-miR-21 was associated with the p53 pathway, and hsa-miR-326 was involved in the FoxO pathway. Our study sheds light on the importance of the mRNA–miRNA–lncRNA network, including important nodes that may play critical roles in HCC pathogenesis.

Previous bioinformatics analyses focus primarily on differentially expressed genes or DEmiRNAs between disease states and controls [15]. However, an increasing amount of literature consider data from the whole transcriptome for discovery of mechanisms of cancer progression. Donahue et al. [17] developed a method to identify genes that predict prognosis of patients with pancreatic cancer by analyzing mRNA and miRNA expression patterns. Herein, we report a new biomarker screening method by integrating these data and constructing a regulatory network, which will be beneficial for further mechanistic exploration of HCC disease. We integrated miRNA–mRNA, miRNA–lncRNA, and mRNA–lncRNA interacting pairs by identifying differentially expressed RNAs and constructed a novel mRNA–miRNA–lncRNA complex regulatory network associated with HCC prognosis. A few studies have constructed mRNA–miRNA–lncRNA regulatory networks in HCC [18]. In most cases, these studies used only the protein interaction database to construct the network *in silico*, an approach that does not include gene expression data. Our study provides an improved network based on the following perspectives. First, we explored the correlation for both miRNA–mRNA and miRNA–lncRNA to identify new biomarkers that predict HCC prognosis. Second, our mRNA co-expression network provided information on genes with relatively unknown functions in correlation with specific biological processes, helping to prioritize candidate genes for functional validation in HCC [19]. Indeed, we identified genes that were poorly studied or characterized but may play important roles in HCC. Although gene co-expression networks do not usually provide information about causality, our co-expression network analysis will guide us in identifying important regulatory genes involved in different phenotypes of HCC. Therefore, it is important to construct both a co-expression network and an interaction network for biomarker discovery in HCC.

Three mRNAs (*CHST4*, *SLC22A8*, and *STC2*) and two miRNAs (hsa-miR-326 and hsa-miR-21) were significantly correlated with HCC patient survival in our study. This result may provide new insights into investigating cancer biomarkers. Our data suggested that these five candidates were prognostic biomarkers, which was further confirmed by our clinical data (Figure 6). In fact, previous studies support the roles of these five molecules in HCC.

*CHST4* encodes an *N*-acetylglucosamine 6-*O* sulfotransferase, a carbohydrate sulfotransferase that catalyzes sulfation reactions [20]. Carbohydrate sulfation is widespread in the extracellular matrix and on cell surfaces [21]. *CHST4* is critical for the biosynthesis of MECA-79-sulfated glycans in the apical membranes of small-sized intrahepatic bile ducts as well as in the cholangiolocellular carcinoma (CoCC) cells, and may serve as a useful marker for CoCC [22], [23]. In accordance with this hypothesis, *CHST4* was up-regulated in cancer tissues and associated with survival of patients with CoCC. *CHST4* is also up-regulated in paediatric precursor-B acute lymphoblastic leukaemia and colonic mucinous adenocarcinoma [24], [25]. Furthermore, *CHST4* is considered as a potential biomarker for early-stage uterine cervical and corpus cancers [26]. Thus, *CHST4* may be involved in HCC progression and prognosis through *O*-glycan processing, which awaits future investigation.

*SLC22A8* encodes a protein in the solute carrier (SLC) family. SLC transporters transfer a wide range of substrates, including inorganic ions, metal ions, saccharides, lipids, amino acids, peptides, proteins, and xenobiotics, across biological membranes [27]. Previous studies suggest that SLC transporters may confer sensitivity to anticancer drugs [28], [29]. For example, the organic cation transporters SLC22A1, SLC22A2, and SLC22A3 enhance cell sensitivity to platinum drugs [30]. SLC22A8 mediates the excretion of many endogenous substances and xenobiotics. Genetic variation of *SLC22A8* could alter its activity, affecting elimination of certain metabolites [31]. SLC22A8 overexpression in lymphoma and the high affinity of SLC22A8 for bendamustine are associated with cytostatic efficiency of bendamustine in lymphoma cells [32]. To our knowledge, the role of SLC22A8 in HCC has not been investigated. Given its role in cancer drug resistance, dysregulated *SLC22A8* expression may be a key risk factor for drug resistance of HCC.

Stanniocalcin (STC) is a family of secreted glycoprotein hormones, consisting of STC1 and STC2, and was discovered in the corpuscles of Stannius [33]. STC2 is involved in calcium and phosphate homeostasis and implicated in the progression of cancer [34]. STC2 is a marker of poor prognosis in patients with gastric cancer or renal cell carcinoma [35], [36]. Similarly, we found that STC2 was significantly correlated with the survival of patients with HCC. More recently, Chen et al. [37] have demonstrated that STC2 functions in the tumorigenesis and progression of colorectal cancer by promoting epithelial-mesenchymal transition, a phenotypic conversion strongly linked with cancer metastasis. Indeed, previous research has shown that STC2 expression correlates with HCC patient prognosis [38], which is consistent with our results. Therefore, STC2 is a promising prognostic biomarker in patients with HCC.

As a class of small non-coding RNAs, miRNAs regulate expression of approximately one-third of protein-coding genes by post-transcriptional mechanisms [39]. Alterations in miRNAs occur during cancer progression [40]. Many miRNAs may serve as accurate predictors of prognosis in human cancers, including HCC [41]. In the present study, hsa-miR-326 and hsa-miR-21 were significantly correlated with HCC patient survival. Previous reports have shown that miR-326 might serve as a tumor suppressor via KRAS or TWIST1 suppression in solid cancers [42], [43]. Consistently, we found that has-miR-326 expression was down-regulated in HCC compared with normal tissue samples in the TCGA database (Figure S2A). Thus, hsa-miR-326 down-regulation may play an important role in HCC tumorigenesis. In addition to its role in cancer pathogenesis, miR-326 is also involved in pro-tumor immunity, in part by promoting TH-17 differentiation [44], [45]. Moreover, miR-326 plays a key role in regulating TGF-β1 expression [46], which can be tumor-promoting or -suppressive in HCC [47]. miR-326 may have a Jekyll and Hyde role in HCC. For example, miR-326 may promote the early stages of HCC carcinogenesis associated with poor survival. However, chronic and long-term miR-326 overexpression may predict better response to treatment in patients with HCC. Moreover, our study suggests that miR-326 is involved in the FoxO signaling pathway. FoxO integrates transcription among pathways regulating proliferation, differentiation, survival, and angiogenesis, and is normally associated with tumor suppressor activity [48], [49]. miR-326 may modulate HCC progression by regulating FoxO signaling; however, the underlying mechanism remains to be elucidated. Although miR-326 overexpression is an important prognosis factor in several cancer types, including gastric cancer and pancreatic ductal adenocarcinoma [50], [51], it was not associated with patient survival in colon or pancreatic adenocarcinomas based on TCGA data (Figure S2B and C). Therefore, the role of miR-326 in cancer may be context-dependent.

miR-21 is a well-characterized miRNA implicated in many types of malignancies, including HCC [52]. miR-21 promotes cell proliferation and inhibits apoptosis by suppressing tumor suppressor genes, including *Bcl-2* [53]. Importantly, Shi et al. [54] have found that high expression of miR-21 correlates with worse 3-year or 5-year survival in HCC patients through Cox regression analysis, consistent with our results. Furthermore, miR-21-5p is involved in the regulation of the p53 signaling pathway. p53 is an internal sentinel for DNA damage and certain types of cellular stress, and can induce cell senescence, death, or cell cycle arrest [55]. p53 signaling contributes to hepatocarcinogenesis by regulating cell proliferation and cell apoptosis [56]. Altogether, these results suggest that miR-21 is a potential prognostic factor in HCC. Ongoing research will improve our understanding of the mechanisms through which miR-21-5p regulates p53 signaling in HCC.

## Conclusion

Our study identifies a novel mRNA–miRNA–lncRNA regulatory network associated with the survival of patients with HCC. Five key molecules (*CHST4*, *SLC22A8*, *STC2*, hsa-miR-326, and hsa-miR-21) serve as potential prognostic markers for HCC potentially through regulation of p53 and FoxO signaling patheways. Further mechanistic studies focusing on these genes and miRNAs are needed to understand the underlying causes of hepatocarcinogenesis.

## Materials and methods

### Data collection

HCC data were downloaded from the TCGA liver hepatocellular carcinoma (TCGA-LIHC) data collection (https://gdc-portal.nci.nih.gov/), which includes clinical information for 377 samples (Table S1). The mRNA and miRNA expression data were also downloaded from the same project. The miRNA-seq data were collected from 372 cancer tissues and 50 normal tissues, and the RNA-seq data were obtained from 371 cancer tissues and 50 cancer-adjacent tissues.

### Differential expression and functional enrichment analyses

Relations of mRNAs and lncRNAs were annotated as stated by the HUGO Gene Nomenclature Committee (HGNC) (https://www.genenames.org/), which includes 19,180 coding genes and 3860 lncRNAs. The edgeR package in R project was used to analyze and identify DEmRNAs and DEmiRNAs as well as DElncRNAs. The thresholds of differential expression analysis were set as adjusted *P* < 0.05 and |Log_2_ fold change| > 1.0. GO and KEGG analyses were performed using clusterProfiler in R [57]. Biological processes and pathways with adjusted *P* < 0.05 were considered significant.

### mRNA co-expression analysis

WGCNA is a systems biology method used to identify clusters of highly correlated genes [58]. In this study, to further explore the interaction between mRNAs in biological networks, we used WGCNA to analyze the co-expressed mRNAs based on mRNA expression profile. Briefly, from the mRNA dataset, we selected all mRNAs with *P* < 0.05 as the reference dataset. Then, we performed survival analysis for each mRNA in the mRNA dataset based on the processed clinical information and experimental group data in the mRNA expression profile to obtain *P* values. Finally, module mining and correlation analysis were conducted using the WGCNA package in R. The degree (k) of each mRNA in modules and Cox regression *P* value between mRNA and sample survival time were calculated to identify the correlation between k and −Log_10_ *P* value.

### Construction of the DEmiRNA–mRNA regulatory network

The miRNA–mRNA pairs were first downloaded from the miRTarBase (https://mirtarbase.mbc.nctu.edu.tw) and miRecords (https://c1.accurascience.com/miRecords/) databases, both of which contain extensive information about experimentally verified miRNA–target interactions [59], [60]. Then, the DEmiRNA–mRNA pairs were selected to construct the DEmiRNA–mRNA regulatory network using Cytoscape [16]. The degree and betweenness centrality of nodes in the network were calculated to analyze their topological properties. The enriched pathways of DEmRNAs and DEmiRNAs were analyzed using clusterProfiler.

### Construction of the mRNA–miRNA–lncRNA complex regulatory network

miRcode (https://www.mircode.org) is a comprehensive searchable map of putative miRNA target sites across the complete GENCODE annotated transcriptome [61]. The sRNA target Base (starBase; https://starbase.sysu.edu.cn/) database was developed to systematically identify protein–RNA interaction networks from CLIP-Seq datasets [62]. We integrated the miRNA–lncRNA and mRNA–lncRNA interacting pairs from miRcode and starBase and then screened out the DEmiRNA–lncRNA pairs and DEmRNA–lncRNA pairs, respectively. Based on the identified RNA–RNA interacting pairs, an mRNA–miRNA–lncRNA complex regulatory network was constructed. Subsequently, according to the expression levels of DEmiRNAs and DEmRNAs and the sample clinical data, we conducted Cox analysis to obtain genes significantly correlated with patient survival in HCC. Finally, the mRNAs and miRNAs with Cox *P* < 0.05 were selected, and their subnetworks were extracted for GO function enrichment analysis using the ClueGO plug-in in Cytoscape [16].

### Follow-up and prognosis evaluation

Retrospective analysis was performed on 258 patients who received curative resection. HCC was defined according to American Association for the Study of Liver Diseases guidelines and was validated by pathological tests. All enrolled patients were followed every 2 months during the first year after surgery and every 6 months afterwards. Patients received chest X-ray, abdominal ultrasonography, and serum AFP tests every 6 months. If a patient was suspected of having a recurrence, ccomputerized tomography (CT) or magnetic resonance imaging (MRI) was used to verify the recurrence or distal metastasis. Follow-up evaluations began in January 2013 and ended in April 2017.

### IHC staining and RT-PCR assays

Antigen retrieval of tissue microarray slides was performed by pressure-cooking in 0.08% citrate buffer for 20 min. Primary antibodies used were anti-CHST4 (1:200; Catalog No. 66623-1-Ig, Proteintech, Manchester, UK), STC2 (1:200; Catalog No. 10314-1-AP, Proteintech), and SLC22A8 (1:200; Catalog No. ab247055, Abcam, Cambridge, UK). Normal control tissues were used to determine the optimal sensitivity and specificity of antibody dilutions. Negative controls were processed with no primary antibody. Results of IHC staining were evaluated by two independent pathologists who were blinded to patient information. Any disagreements were resolved by discussion, and, when necessary, a third reviewer was consulted. Staining extent was scored as 0, 1, 2, 3, or 4, according to the percentages of immunoreactive tumor cells (0%, 1%–5%, 6%–25%, 26%–75%, 76%–100%, respectively). Staining intensity was defined and scored as negative (0), weak (1), or strong (2). A score ranging from 0 to 8 was calculated by multiplying the staining extent score with the intensity score, resulting in a low (0–4) level or a high (6–8) level value for each specimen.

To evaluate the expression patterns of the selected mRNAs/miRNAs, 258 HCC tissues were collected from HCC patients who underwent curative resection at Zhongshan Hospital, Shanghai, China. Another 28 HCC and paired adjacent normal liver tissues were also collected to compare gene expression in HCC and normal tissues. Total RNA was extracted from frozen HCC tissues with RNeasy Mini Kit (Qiagen, Shanghai, China). QuantiTect Reverse Transcription Kit (Qiagen) was used for reverse transcription of mRNA. miRNA First Strand cDNA Synthesis Kit (Sangon Biotech, Shanghai, China) was used for reverse transcription of miRNA. RT-PCR was performed by Taqman microRNA assays (ThermoFisher Scientific, Shanghai, China). *U6* and *GAPDH* served as internal controls for PCR assays. The following primers were used: *STC2*-F (5ʹ-CCAGGGCAAGTCATTCATCA-3ʹ) and *STC2*-R (5ʹ-TCACGAGGTCCACGTAGGGT-3ʹ); *CHST4*-F (5ʹ-GGAGGACCAACCCTACTATGTG-3ʹ) and *CHST4*-R (5ʹ-CTTGCCTCGGGTGATGTTAT-3ʹ); *SLC22A8*-F (5ʹ-CCTGGCCTGGTTTGCTAC-3ʹ) and *SLC22A8*-R (5ʹ-GAACTTGGCTGGGACATCGAC-3ʹ); *GAPDH*-F (5ʹ-ATGGGGAAGGTGAAGGT-3ʹ) and *GAPDH*-R (5ʹ-AAGCTTCCCGTTCTCAG-3ʹ); miR-326 (5ʹ-CCTCTGGGCCCTTCCTCC-3ʹ); miR-21 (5ʹ-ccgcgTAGCTTATCAGACTGATGTTGA-3ʹ). All experiments were conducted in triplicate.

## Ethical statement

This study was supervised and approved by the ethics committee of Zhongshan Hospital of Fudan University, China. Informed written consents were obtained from participants in accordance with the guidelines of the ethics committee.

## Competing interests

The authors have no competing interests to declare.

## CRediT authorship contribution statement

**Bo Hu:** Conceptualization, Methodology. **Xiaolu Ma:** Conceptualization. **Peiyao Fu:** Writing – original draft. **Qiman Sun:** . **Weiguo Tang:** Data curation. **Haixiang Sun:** Writing – review & editing. **Zhangfu Yang:** Data curation. **Mincheng Yu:** Data curation. **Jian Zhou:** Writing – review & editing. **Jia Fan:** Writing – review & editing. **Yang Xu:** Supervision, Project administration, Funding acquisition.

## Supplementary material

Supplementary Figure S1 **The selected genes related to the prognosis of HCC** The Kaplan-Meier survival curves (**A**) and ROC curves (**B**) of for *CDH6*, *CXCL1*, *DNER*, *IL20RB*, *PROK1*, *SBSN*, and *TCN1* significantly correlated with HCC patient survival, with high expression predicting worse prognosis.

Supplementary Figure S2 **The expression of miR-326 in TCGA A.** miR-326 expression was down-regulated in HCC compared to normal tissues from TCGA database. **B.** miR-326 was not associated with patient survival in COAD according to TCGA data. **C.** miR-326 was not associated with patient survival in PAAD according to TCGA data. COAD, colon adenocarcinoma; PAAD, pancreatic adenocarcinoma.

Supplementary Table S1 **Clinical information of 377 samples from TCGA-LIHC data collection**
