# Supplementary figures and images for: The mRNA–miRNA–lncRNA Regulatory Network and Factors Associated with Prognosis Prediction of Hepatocellular Carcinoma
Source: Genomics Proteomics Bioinformatics. 2021 Mar 17;19(6):913–25. doi: 10.1016/j.gpb.2021.03.001 (PMC9402792; doi:10.1016/j.gpb.2021.03.001)

**A**

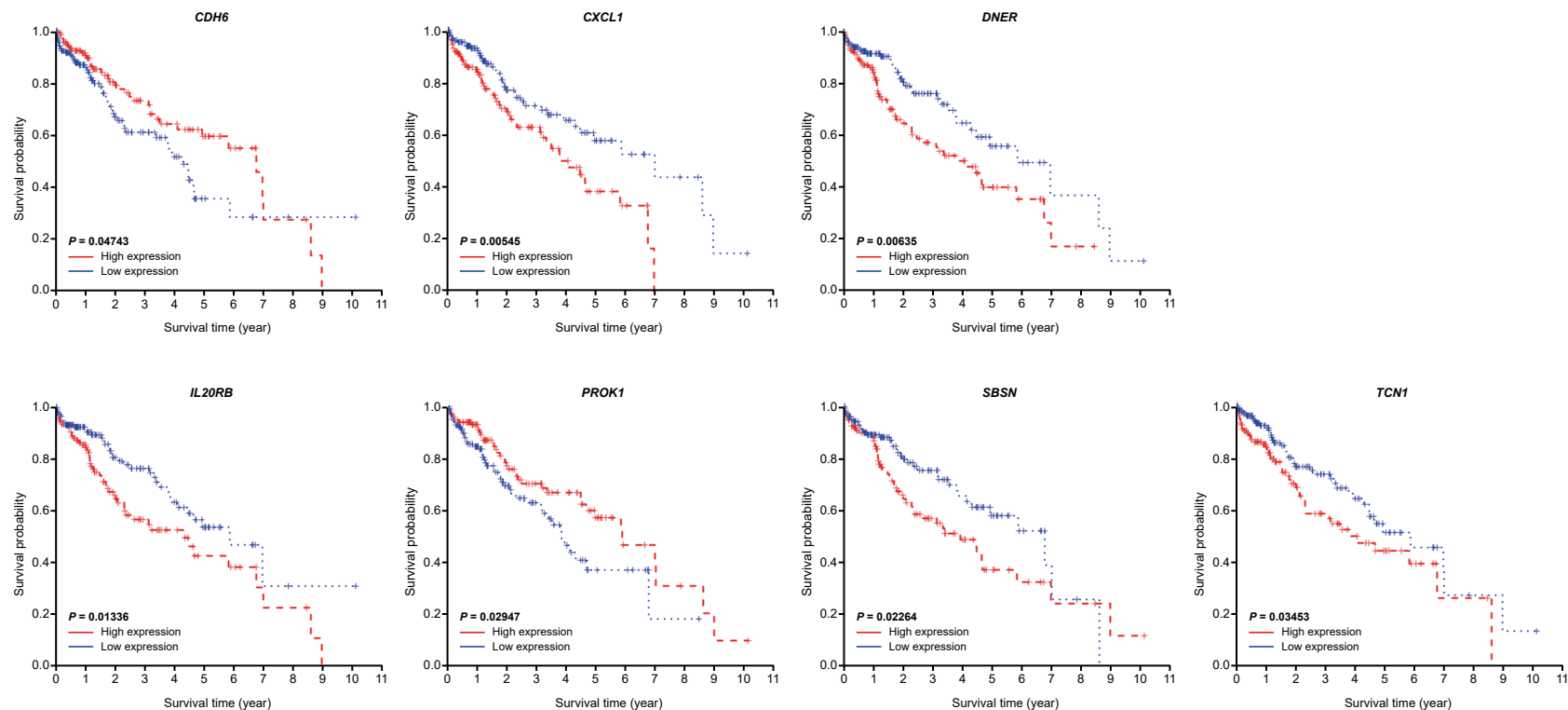

**B**

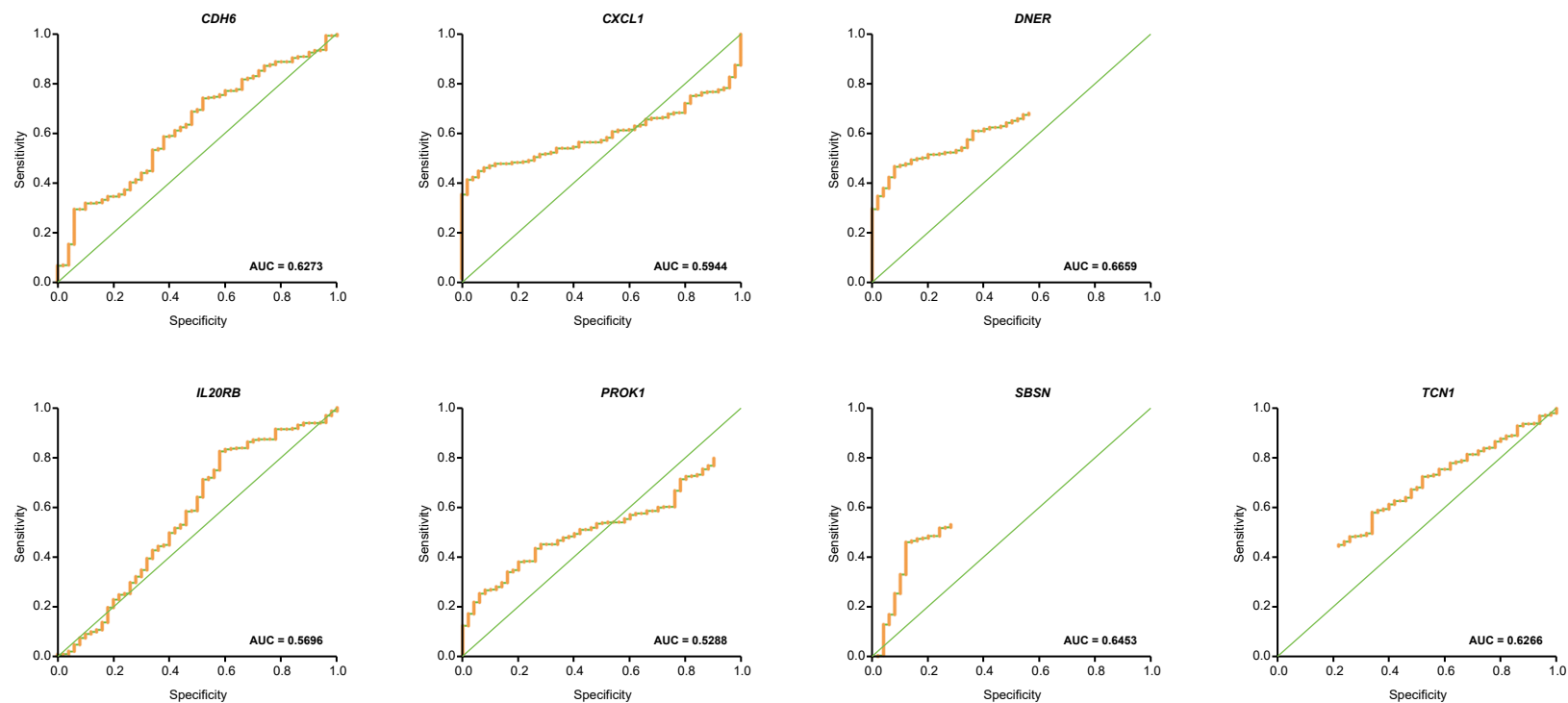

Supplement: Supplementary Figure S1 — The selected genes related to the prognosis of HCC The Kaplan-Meier survival curves (A) and ROC curves (B) of for CDH6, CXCL1, DNER, IL20RB, PROK1, SBSN, and TCN1 significantly correlated with HCC patient survival, with high expression predicting worse prognosis. [file mmc1.pdf]

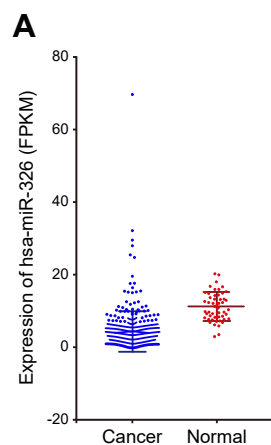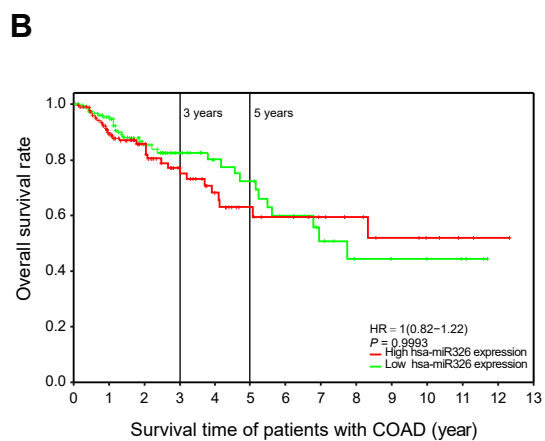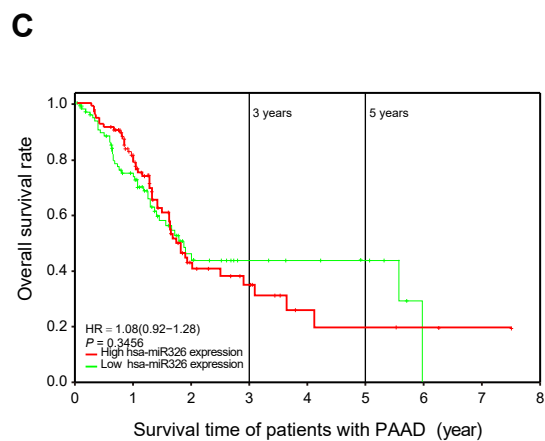

Supplement: Supplementary Figure S2 — The expression of miR-326 in TCGA A. miR-326 expression was down-regulated in HCC compared to normal tissues from TCGA database. B. miR-326 was not associated with patient survival in COAD according to TCGA data. C. miR-326 was not associated with patient survival in PAAD according to TCGA data. COAD, colon adenocarcinoma; PAAD, pancreatic adenocarcinoma. [file mmc2.pdf]
